# Perinatal Smoking Patterns From Preconception to 1-Year Post Partum

**DOI:** 10.1001/jamanetworkopen.2024.54974

**Published:** 2025-01-17

**Authors:** Heidi Allen, Jamie Daw

**Affiliations:** 1 School of Social Work, Columbia University, New York, New York; 2 Department of Health Policy and Management, Columbia University Mailman School of Public Health, New York, New York

## Abstract

This cohort study assesses rates of maternal smoking in the United States during the perinatal period overall and by age, race and ethnicity, insurance type, and geography.

## Introduction

Smoking cessation is a major focus of prenatal care, offering the opportunity to prevent smoking-related pregnancy complications, promote long-term abstinence, and reduce secondhand exposure for infants and children.^1,2^ Recent representative multistate survey data from 2021 show that most people who smoke (56%) quit during pregnancy and the early postpartum smoking rate is half of the prepregnancy rate (7% compared with 12%).^3^ Population-level data on longer term relapse after pregnancy is limited. The objective of this study was to estimate perinatal smoking patterns from preconception through 1-year post partum.

## Methods

This cohort study was approved by the institutional review board at all study jurisdictions and Columbia University. This cohort study followed the Strengthening the Reporting of Observational Studies in Epidemiology (STROBE) reporting guideline; data sources and variables are available in the eMethods in Supplement 1. Informed consent was obtained electronically or by phone, depending on mode of survey administration. We used longitudinal individually linked data from birth certificates (BC), the Pregnancy Risk Assessment Monitoring System (PRAMS), and the Postpartum Assessment of Health Survey (PAHS) for a representative sample of live births in 2020 in 6 states (Kansas, Michigan, New Jersey, Pennsylvania, Utah, Virginia) and New York City. We measured self-reported maternal smoking at 4 time points: preconception (3 months before pregnancy; PRAMS and BC), during pregnancy (last 3 months of pregnancy; PRAMS and BC), early post partum (mean [SD], 3.9 (1.2) months after childbirth; PRAMS) and late post partum (mean [SD], 13.1 [0.8] months after childbirth; PAHS) (eTable 1 in Supplement 1). We examined longitudinal smoking patterns and differences by age, race and ethnicity, insurance at birth, and geography (eTable 2 in Supplement 1). We also measured whether respondents reported discussing smoking cessation during prenatal and postpartum visits (eTable 3 in Supplement 1). We weighted all estimates to be representative of 2020 live births in the participating jurisdictions and estimated unadjusted logistic regression models to conduct statistical comparisons between groups. Two-sided *P* < .05 was considered statistically significant. Data were analyzed from January to November 2024 using Stata version 18 (StataCorp).

## Results

The sample included 4547 postpartum individuals (1247 aged 25 to 29 years [26.4%]; 1504 aged 30 to 34 years [32.4%]; 621 Black individuals [14.8%]; 2609 White individuals [54%]). Of these individuals, 2879 (58.4%) were commercially insured and 3970 (89.1%) lived in an urban or suburban area. The prevalence of smoking was 14.6% (95% CI, 13.1%-16.2%) preconception, 7.2% (95% CI, 6.1%-8.5%) during pregnancy, 7.0% (95% CI, 5.9%-8.4%) early post partum, and 11.5% (95% CI, 10.1%-13.0%) late post partum (Table). We identified 6 mutually exclusive perinatal smoking patterns: never smoked (83.5%), always smoked (5.1%), persistent pregnancy quitting (3.6%), postpartum relapse (3.8%), initiated post partum (1.9%), and quit post partum (1.0%).

**Table.** Perinatal Smoking Patterns by Maternal Sociodemographic Characteristics

Sample characteristics	Sample size, No. (weighted %)	Perinatal smoking patterns weighted, % (95% CI)^a^					
		Never smoked	Always smoked	Persistent prenatal quitting	Relapsed post partum	Initiated post partum	Quit post partum
Overall	4547	83.5 (81.7-85.1)	5.1 (41.-6.3)	3.6 (2.9-4.5)	3.8 (3.0-4.9)	1.9 (1.4-2.6)	1.0 (0.6-1.6)
Age, y							
18-24^b^	748 (19.3)	78.3 (72.9-82.8)	5.2 (3.0-8.8)	3.5 (2.0-6.0)	6.8 (4.4-10.4)	3.8 (2.4-5.9)	0.9 (0.3-2.4)
25-29	1247 (26.4)	82.6 (78.2-86.2)	4.9 (3.1-7.5)	4.1 (2.7-6.4)	3.4 (2.1-5.5)	2.5 (1.4-4.5)	0.8 (0.3-1.7)
30-34	1504 (32.4)	85.5 (82.1-88.3)^c^	5.8 (4.2-8.0)	2.5 (1.6-3.8)	3.6 (2.2-5.8)	1.1 (0.5-2.3)^c^	1.2 (0.5-2.8)
≥35	1048 (21.9)	86.2 (82.9-89.0)^d^	4.1 (2.6-6.5)	4.6 (2.9-7.1)	2.1 (1.3-3.3)^d^	0.8 (0.4-1.9)	1.1 (0.3-3.3)
Race or ethnicity^e^							
Black	621 (14.8)	82.2 (77.0-86.5)	5.3 (2.6-10.8)	3.3 (1.7-6.5)	2.3 (1.3-4.0)^c^	4.8 (2.8-8.0)^f^	1.0 (0.5-2.2)
Hispanic or Latinx	817 (18.8)	90.4 (86.9-93.0)^f^	1.3 (0.7-2.4)^f^	2.6 (1.5-4.7)	2.3 (1.4-3.9)^c^	2.0 (1.1-3.7)	1.1 (0.3-3.8)
Native American or Alaska Native	47 (0.8)	68.4 (39.0-88.0)	2.6 (0.8-8.5)	10.2 (1.9-39.3)	6.3 (0.7-39.8)	NA	8.5 (1.2-41.6)
White^b^	2609 (54.0)	80.1 (77.4-82.6)	7.1 (5.6-8.9)	4.2 (3.1-5.5)	5.1 (3.6-7.1)	1.0 (0.6-1.8)	0.9 (0.5-1.7)
Additional groups^g^	385 (10.3)	93.1 (89.0-95.8)^f^	0.8 (0.3-2.3)	2.4 (0.9-5.9)	0.7 (0.3-1.8)^d^	2.0 (0.7-6.1)	0.8 (0.1-4.9)
Multiple races or ethnicities	66 (1.2)	74.4 (57.0-86.5)	3.6 (1.2-10.4)	1.1 (0.2-5.4)	15.7 (6.8-32.2)^c^	5.2 (0.9-24.4)	NA
Insurance at birth							
Commercial^b^	2879 (58.4)	90.0 (87.9-91.7)	1.5 (1.0-2.1)	3.5 (2.6-4.6)	2.6 (1.8-3.7)	1.0 (0.6-1.8)	0.6 (0.3-1.5)
Medicaid	1526 (38.3)	73.4 (69.6-76.8)^f^	10.5 (8.1-13.6)^f^	3.9 (2.7-5.8)	5.5 (3.9-7.5)^d^	3.4 (2.3-5.0)^d^	1.6 (0.9-2.7)
Uninsured	141 (3.2)	85.5 (70.2-93.6)	5.6 (1.7-16.8)	0.9 (0.1-5.3)	7.0 (2.1-21.4)	0.3 (0.1-1.0)	0.8 (0.1-5.9)
Geography							
Urban or suburban^b^	3970 (89.1)	84.2 (82.4-85.9)	4.2 (3.3-5.3)	3.5 (2.8-4.4)	3.7 (2.9-4.9)	2.0 (1.5-2.7)	1.0 (0.6-1.7)
Rural	525 (9.7)	76.7 (68.5-83.3)^c^	12.4 (7.5-20.0)^f^	4.0 (1.9-8.3)	4.5 (2.2-9.0)	1.4 (0.5-3.8)	0.7 (0.1-5.0)

^a^ Perinatal smoking patterns based on reported smoking at 4 time points: preconception (3 months prior to pregnancy), pregnancy (last 3 months of pregnancy), early post partum (mean of 4 months after childbirth) and late post partum (mean of 13 months after childbirth). Other perinatal smoking patterns represented 1.1% of the sample and are not shown.

^b^ Reference group.

^c^ *P* < .05.

^d^ *P* < .01.

^e^ Race and ethnicity is self-reported and multiple race and ethnicity is defined as selecting more than 1 response option.

^f^ *P* < .001.

^g^ Additional groups include Asian, Native Hawaiian, Pacific Islander, Southwest Asian, Middle Eastern, or North African.

Younger people (ie, aged 18 to 24 years) were less likely than people aged 30 years or older to report never smoking and more likely to relapse post partum (Table). White individuals had the highest rates of always smoking (7.1%; 95% CI, 5.6%-8.9%) and postpartum relapse (5.1%; 95% CI, 3.6%-7.1%); however, Black individuals were more likely to initiate smoking post partum (4.8%; 95% CI, 2.8%-8.0% vs 1.0%; 95% CI, 0.6%-1.8%; *P* < .001). Always smoking rates were nearly 3 times higher for rural compared with urban or suburban individuals (12.4%; 95% CI, 7.5%-20% vs 4.2%; 95% CI, 3.3%-5.3%; *P* < .001). Medicaid enrollees were more likely to be always smokers (10.0%; 95% CI, 8.1%-13.6% vs 1.5%; 95% CI, 1.0%-2.1% commercially-insured; *P* < .001) and to quit and resume (5.5%; 95% CI, 3.9%-7.5% vs 2.6%; 95% CI, 1.8%-3.7%; *P* < .01). Smoking rates were higher for Medicaid-insured individuals at all time points with a 11% decline from preconception to late post partum, compared with a 37% decline among those with commercial insurance (Figure). Nearly all respondents (93.7%; 95% CI, 92.7%-94.6%) reported discussing smoking during prenatal care visits compared with only 47.9% (95% CI, 45.8%-50.1%) during the routine postpartum check-up.

**Figure.**
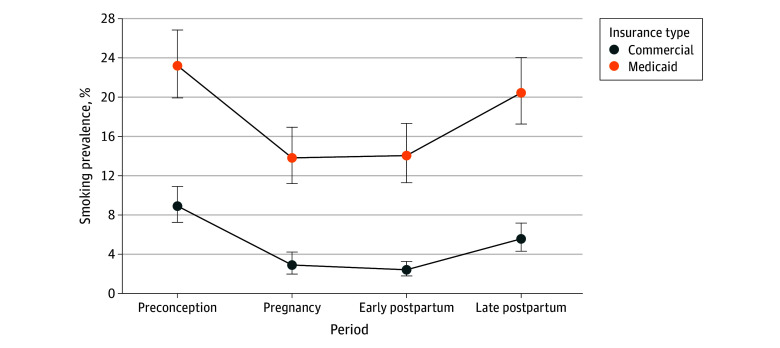
Trends in Perinatal Smoking by Insurance Type at Birth Survey-weighted smoking prevalence based on reported smoking at 4 time points: preconception (3 months prior to pregnancy), pregnancy (last 3 months of pregnancy), early post partum (mean of 4 months after childbirth), and late post partum (mean of 13 months after childbirth). Uninsured at birth not shown due to small sample size (n = 141). Difference between commercial and Medicaid is statistically significant (*P* < .05) at all 4 time points.

## Discussion

These findings suggest that there is substantial opportunity to prevent smoking relapses and initiation in the postpartum year, which would promote the short- and long-term health of families. Individuals who are younger, Medicaid-insured, identify as White or Black, and live in rural areas should be of particular focus. Recent extensions of postpartum Medicaid coverage offer a new platform to promote long-term smoking cessation; however, state Medicaid coverage for smoking cessation treatment varies with documented barriers to access.^4^ Smoking counseling should be a key component of routine postpartum care, and prioritized during transitions from pregnancy to primary care. Limitations include self-reported outcomes and small samples for some subgroup comparisons.

## Notes

Supplement 1.
eMethods.
**eTable 1.** Smoking Outcome Data Sources and Definitions **eTable 2.** Smoking Pattern Definitions **eTable 3.** Smoking Counseling Data Sources and Variable Definitions

Supplement 2.
Data Sharing Statement

## References

[1] The health consequences of smoking—50 years of progress: a report of the Surgeon General: (510072014-001). United States Surgeon General. Accessed December 5, 2024. https://www.ncbi.nlm.nih.gov/books/NBK179276/

[2] Jones M, Lewis S, Parrott S, Wormall S, Coleman T. Re-starting smoking in the postpartum period after receiving a smoking cessation intervention: a systematic review. Addiction. 2016;111(6):981-990. doi:10.1111/add.13309 26990248 PMC6680353

[3] Kipling L, Bombard J, Wang X, Cox S. Cigarette smoking among pregnant women during the perinatal period: prevalence and health care provider inquiries: pregnancy risk assessment monitoring system, United States, 2021. MMWR Morb Mortal Wkly Rep. 2024;73(17):393-398. doi:10.15585/mmwr.mm7317a2 38696343 PMC11065467

[4] DiGiulio A, Tynan MA, Schecter A, Williams KS, VanFrank B. State Medicaid coverage for tobacco cessation treatments and barriers to accessing treatments: United States, 2018-2022. MMWR Morb Mortal Wkly Rep. 2024;73(14):301-306. doi:10.15585/mmwr.mm7314a2 38602885 PMC11008788

