# Errors in Table

**DOI:** 10.1001/jamanetworkopen.2025.3405

**Published:** 2025-02-28

In the Original Investigation titled “Perinatal Smoking Patterns From Preconception to 1-Year Post Partum,”^1^ published January 17, 2025, there were formatting errors in the Table. The data columns for the perinatal smoking patterns were shifted one column to the left (eg, the data for the never smoked category were under the column heading for sample size and the always smoked data were under the never smoked column heading). The correct sample size data were omitted. The data in the sample size column were repeated in the quit post partum column. The row headings for Native American or Alaska Native, White, and additional groups were aligned with the incorrect rows. This article has been corrected.^1^
