# Supplementary material for: Perinatal Smoking Patterns From Preconception to 1-Year Post Partum
Source: JAMA Netw Open. 2025 Jan 17;8(1):e2454974. doi: 10.1001/jamanetworkopen.2024.54974 (PMC11742529; doi:10.1001/jamanetworkopen.2024.54974)
Supplement: Supplement 1. — eMethods. eTable 1. Smoking Outcome Data Sources and Definitions eTable 2. Smoking Pattern Definitions eTable 3. Smoking Counseling Data Sources and Variable Definitions [file jamanetwopen-e2454974-s001.pdf]

Supplemental Online Content

Allen HL, Daw JR. Perinatal Smoking patterns from preconception to 1 year postpartum. *JAMA Netw Open*. 2025;8(1):e2454974. doi:10.1001/jamanetworkopen.2024.54974

eMethods.
eTable 1. Smoking Outcome Data Sources and Definitions
eTable 2. Smoking Pattern Definitions
eTable 3. Smoking Counseling Data Sources and Variable Definitions

This supplemental material has been provided by the authors to give readers additional information about their work.

© 2025 Allen HL et al. *JAMA Network Open*.

eMethods
Study data sources

Survey Overview: The Postpartum Assessment of Health Survey (PAHS) is a follow-up survey to the CDC Pregnancy Risk Assessment Monitoring System (PRAMS). The 2020 PAHS included six states (KS, MI, NJ, PA, UT, VA) and New York City. PAHS jurisdictions were selected for having a large PRAMS sample size, consistently meeting the CDC PRAMS response rate thresholds, and willingness and capacity to collaborate with the research team. The 2020 PAHS sampling frame included all 2020 PRAMS respondents (i.e. those who had a live birth in 2020 and were randomly sampled for PRAMS in the participating jurisdictions) who opted-in (MI) or did not opt-out (other 6 jurisdictions) of being recontacted for PAHS. All respondents in the sampling frame were contacted by phone, email and mail from 12-14 months after their live birth with the opportunity to participate in PAHS. Participants were required to provide verbal or written consent to participation and a $45 gift card was provided for survey completion.

Measures: The 2020 PAHS questionnaire contained 108 core questions about health and well-being; social needs; and health care in the first year postpartum, largely using validated question designs from other population health surveys. PAHS was offered in both English and Spanish. PAHS responses were linked on an individual-level to birth certificate variables and PRAMS survey responses. eTable 1 shows the data source of each smoking outcome variable. We defined smoking as any self-reported smoking across any of the variables measuring smoking at each time point. Notably, the early postpartum smoking measure is based on a question that asks only about cigarette smoking, whereas e-cigarettes and electronic nicotine products are included for the other three time points. Thus, the early postpartum measure may underestimate smoking compared to other time points. Comparing the preconception and pregnancy time points where cigarette and e-cigarette use is measured separately suggests that not including e-cigarette use results in an approximately 10% lower estimate of smoking. eTable 2 and eTable 3 provide the definitions and data sources for perinatal smoking patterns and smoking counselling outcomes. Maternal sociodemographics were self-reported and measured on PAHS with imputation from PRAMS or the birth certificate for missing values.

Sample Size and Response Rate: The total 2020 PRAMS sampling frame across the 7 sites was N=14,314. Across the 7 sites, 28.9% of PRAMS respondents chose not to be recontacted for PAHS (19% in 6 opt-out sites; 78.9% in opt-in site of Michigan). This resulted in a total PAHS sampling frame of N=6021. Based only on complete responses, the unweighted PAHS response rate relative to the PAHS sampling frame was 76.4% (N=4598). The analysis for this study included only PAHS respondents with complete data for smoking outcomes at all time points (Table A1), N=4547 (98.9%)

Survey Weights: The 2020 PAHS survey weights are designed to generate representative estimates of all live births in 2020 in the 7 participating jurisdictions. The PAHS weights build on the PRAMS survey weights and account for the stratified survey design of PRAMS as well as PRAMS and PAHS nonresponse. The weights are further calibrated on key characteristics including PRAMS sampling strata, maternal age, marital status, race, Hispanic ethnicity, education, and infant birthweight to align with total population values for all live births in the participating jurisdictions.

© 2025 Allen HL et al. *JAMA Network Open*.

eTable 1 Smoking outcome data sources and definitions

Time Point	Data Source	PAHS or PRAMS Survey Question	Birth Certificate
Preconception	PRAMS and Birth Certificate	*In the 3 months before you got pregnant, how many cigarettes did you smoke on an average day?* A pack has 20 cigarettes [SMK63B_A]<br><br>• 41 or more<br>• 21-40 cigarettes<br>• 11-20 cigarettes<br>• 6-10 cigarettes<br>• 1-5 cigarettes<br>• Less than 1 cigarette<br>• I didn't smoke then<br><br>*During the 3 months before you got pregnant, on average, how often did you use e-cigarettes or other electronic nicotine products?* [ECIG_3B_A]<br>• More than once a day<br>• Once a day<br>• 2-6 days a week<br>• 1 day a week or less<br>• I did not use e-cigarettes or other nicotine containing e-vaping products then	*How many cigarettes or packs of cigarettes did you smoke on an average day during each of the following times?* [Open Field]<br>[3 months before pregnancy]
Pregnancy (third trimester)	PRAMS and Birth Certificate	*In the last 3 months of your pregnancy, how many cigarettes did you smoke on an average day?* A pack has 20 cigarettes [SMK63L_A]<br>• 41 or more<br>• 21-40 cigarettes<br>• 11-20 cigarettes<br>• 6-10 cigarettes<br>• 1-5 cigarettes<br>• Less than 1 cigarette<br>• I didn't smoke then<br><br>*During the last 3 months of your pregnancy, on average, how often did you use e-cigarettes or other electronic nicotine products*? [ECIG_3L_A]<br>• More than once a day<br>• Once a day<br>• 2-6 days a week	Same as above<br>[Third 3 months of pregnancy]

© 2025 Allen HL et al. *JAMA Network Open*.

		• 1 day a week or less<br>• I did not use e-cigarettes or other nicotine containing e-vaping products then	
Early Postpartum (mean: 4 months)	PRAMS	*How many cigarettes do you smoke on an average day now?* A pack has 20 cigarettes [SMK6NW_A]<br>• 41 or more<br>• 21-40 cigarettes<br>• 11-20 cigarettes<br>• 6-10 cigarettes<br>• 1-5 cigarettes<br>• Less than 1 cigarette<br>• I don't smoke now	NA
Late Postpartum (mean 13 months)	PAHS	*Since giving birth, did you smoke cigarettes, use nicotine products, or vape/use e-cigarettes every day, some days or not at all?* [bhq_nicotine]<br>• Every day<br>• Some days<br>• Not at all	NA

© 2025 Allen HL et al. *JAMA Network Open*.

eTable 2 Smoking pattern definitions

Smoking Pattern	Definition
Always smokers	Smoking at every time point preconception through late postpartum
Never smokers	No smoking at any time point preconception through late postpartum
Persistent Pregnancy Quitters	Preconception smoking followed by no smoking in pregnancy, early postpartum and late postpartum
Postpartum Relapsers	Preconception smoking, no smoking in pregnancy, and smoking relapse in early and/or late postpartum
Postpartum Initiators	No preconception or pregnancy smoking followed by smoking in early and/or late postpartum
Postpartum Quitters	Preconception and pregnancy smoking followed by no smoking in early and/or late postpartum

eTable 3 Smoking counseling data sources and variable definitions

Time Point	Data Source	PRAMS Survey Question
Prenatal Care	PRAMS	*During any of your prenatal care visits, did a doctor, nurse, or other health care worker ask you any of the things listed below?*<br><br>• *If I was smoking cigarettes*
Postpartum Checkup	PRAMS	*During your postpartum checkup, did a doctor, nurse, or other health care worker do any of the following things?*<br><br>• *Ask me if I was smoking cigarettes*

© 2025 Allen HL et al. *JAMA Network Open*.
