# Supplementary material for: Perinatal Smoking Patterns From Preconception to 1-Year Post Partum
Source: JAMA Netw Open. 2025 Jan 17;8(1):e2454974. doi: 10.1001/jamanetworkopen.2024.54974 (PMC11742529; doi:10.1001/jamanetworkopen.2024.54974)
Supplement: Supplement 2. — Data Sharing Statement [file jamanetwopen-e2454974-s002.pdf]

Data Sharing Statement

Allen. Perinatal Smoking Patterns From Preconception to 1 Year Postpartum. *JAMA Netw Open*. Published January 17, 2025. doi:10.1001/jamanetworkopen.2024.54974

Data
Data available: No

Additional Information
Explanation for why data not available: PRAMS data is available upon request from the CDC. The PAHS data is not available to external researchers due to data use agreements with the participating jurisdictions. Variable definitions are provided in the Online Supplement.
